# Metal implant removal: benefits and drawbacks – a patient survey

**DOI:** 10.1186/s12893-015-0081-6

**Published:** 2015-08-07

**Authors:** Georg Reith, Vera Schmitz-Greven, Kai O. Hensel, Marco M. Schneider, Tibor Tinschmann, Bertil Bouillon, Christian Probst

**Affiliations:** Department of Trauma and Orthopaedic Surgery, Cologne Merheim Medical Center, Witten/Herdecke University, Cologne, Germany; Helios Medical Center Wuppertal, ZBAF, Center for Biomedical Education and Research, Witten/Herdecke University, Witten, Germany

**Keywords:** Metal removal, Implant removal, Metalwork removal, Hardware removal, Complication, Patient satisfaction

## Abstract

**Background:**

Hardware removals are among the most commonly performed surgical procedures worldwide. Current literature offers little data concerning postoperative patient satisfaction. The purpose of our study was to evaluate the patients’ point of view on implant removal.

**Methods:**

We surveyed patients of a German level one trauma center, who underwent hardware removal in 2009 and 2010, with regard to their personal experiences on implant removal. Exclusively, data obtained out of the survey were analyzed.

**Results:**

In 332 patients surveyed, most hardware removals were performed at the ankle joint (21 %) followed by the wrist (15 %). The most frequent indication was a doctor’s recommendation (68 %), followed by pain (31 %) and impaired function (31 %). Patient reported complication rate of implant removal was 10 %. Importantly, after implant removal because of pain or impaired function patients reported an improvement in function (72 %) as well as decreased pain (96 %). 96 % of all responding patients and 66 % of the patients who suffered from subsequent complications would opt for surgical implant removal again.

**Conclusion:**

In summary, despite the challenging and frequently troublesome nature of surgical hardware removal our data contradicts the widely held view that implant removal is often without a positive effect on the patients. These findings may influence the surgeons’ attitude towards implant removal and their day-to-day routine in patient counseling.

**Electronic supplementary material:**

The online version of this article (doi:10.1186/s12893-015-0081-6) contains supplementary material, which is available to authorized users.

## Background

Surgical removal of hardware for internal fixation of fractured bones is one of the most frequently performed orthopedic surgeries in the western world [1]. In 2010, a total of 180,000 hardware removal surgeries were performed in Germany, making it the fourth most common surgical procedure in orthopedic surgery after surgical fracture fixation, arthroscopies and intervertebral disc interventions [2].

There is an ongoing debate concerning the justification of elective surgical implant removal [3–5]. Certainly, the indication for hardware removal is unquestioned in patients with surgical site infection, metal allergy, soft tissue compromise or failure of the osteosynthesis [4]. However, the benefits of relative indications such as intended improvement of function, foreign body or pain sensation, spatial limitation for future surgical procedures or plainly the patient’s desire for hardware removal have not yet sufficiently been proven.

In a study by Hanson in 2008 which surveyed 730 attendees of the AO Principles and Masters Courses of Operative Fracture Treatment in Davos, Switzerland, 380 of 655 surgeons (58 %) did not agree that routine implant removal is necessary and 48 % felt that removal is riskier than leaving the implant *in situ* [6]. This probably was mainly influenced by numerous complications which can occur during and after operative implant removal.

Commonly observed complications after hardware removal are infections, impaired wound healing, refractures, tissue and nerve damage and post-operative bleeding or an incomplete removal. There is some evidence indicating that the postoperative complication rate depends on the specific localization of the implanted material. However, inter-individual differences are significant and published data still lacks consistency [7–12]. Therefore general recommendations cannot yet be established.

Besides the above mentioned medical issues, the socioeconomic impact must be taken into consideration. Hardware removal is cost consuming for both hospitals and health care resources [1]. In addition, others speculated on the influence on a society’s labor force due to postoperative absence from work without being able to quantify this burden in detail [4].

Aim of this study was to evaluate specifically the patient’s subjective point of view regarding surgical hardware removal using a generally understandable and self-explanatory questionnaire. We hypothesized, that patients’ satisfaction after implant removal is low because of the associated complications.

## Methods

A retrospective review was performed of patients undergoing surgical hardware removal in a German level-1 trauma center.

Potential patients were identified by procedure code (OPS-code) for surgical hardware removal without perioperative antibiotic prophylaxis between January 1^st^ 2009 and December 31^st^ 2010. The information on exact implant location, surgery type and whether the operation was performed as an inpatient or outpatient procedure was taken out of the electronic patient chart. Minors with an age of 6 years and older were as well integrated in the study. Excluded were removals of external fixators and patients without proper contact information and whereabouts. Furthermore, subjects with an incomplete survey who could not be contacted by telephone, were not integrated in the study.

In August 2011, patients were requested to fill out a written survey concerning their individual subjective benefits and burdens of surgical implant removal. Subjects were able to respond to the questionnaire via mail, email, telephone or fax. Two weeks after the survey was initially mailed, every patient was contacted by telephone in order to increase the number of participants, additionally to increase the recruitment of possibly dissatisfied or disgruntled patients who did not want to complete the mailed survey. If possible, the survey was filled out directly via telephone. In case of minors the parents were asked to fill out the survey together with their children. The timeframe of a possible participation was 6 months. During these 6 months, patients, who had not answered, were contacted twice according to a standardized protocol. If they still did not answer, they were considered as drop-out.

The 9-item survey was developed by the authors in view of the hypothesis. Included were questions regarding the patients’ perception of the indications for hardware removal, their function and pain before and after implant removal as well as their complications during or after the procedure. Several questions allowed for multiple responses (Additional file 1). Within the item “reason for hardware removal” the answer “doctor’s recommendation” refers to a recommendation of the patient’s family practitioner or outpatient orthopedic specialist to have the hardware removed without a more specific medical/surgical reason. Furthermore, we assessed, whether the patient would opt for the implant removal again. The questionnaire was set in a generally understandable, colloquial format in German avoiding the use of technical terms, medical scores and official pain schemes. In particular, the patient subjectively evaluated questions concerning pain and function as well as complications. There was no follow up examination or a general or comprehensive electronic chart review.

The answers were correlated to demographic data. Answers of parents/minors were not handled differently. All answers were descriptively analyzed using Microsoft Excel®.

All of the patients surveyed were German citizens, speaking the German language fluently and gave their consent for study participation. The study was approved by the ethical committee of Witten-Herdecke University, Germany.

## Results

A total of 565 patients were identified to have undergone hardware removal, of which only 522 had an accurate address in the medical record to which a survey was sent. Of the 522 patients to which a survey was sent, 186 were considered non-responders and excluded, four patients actively refused to participate in a telephone interview. Hence 332 responders were available for analysis (response rate of 64 %). Incomplete surveys were completed by an additional telephone interview. Concerning demographics patients had a mean age of 46.3 years (+/-19.8) (range 6 to 84). Half of the patients (51 %) were men.

**Fig. 1:**
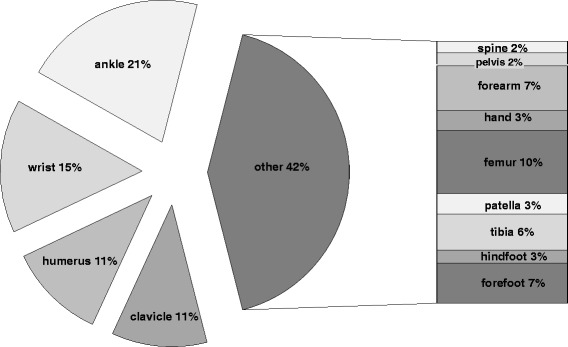
Localizations of surgical hardware removal

**Fig. 2:**
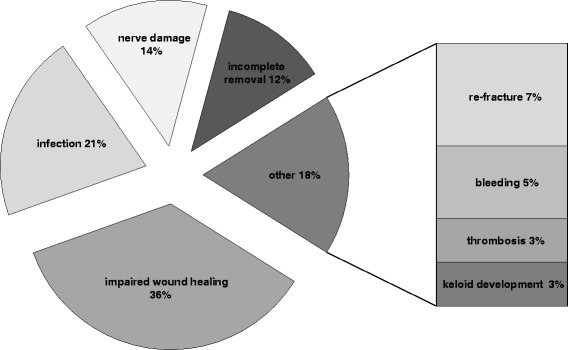
Postoperative complications following surgical hardware removal

**Fig. 3:**
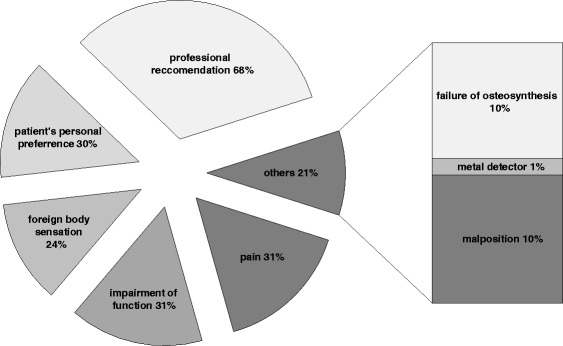
Indications for surgical hardware removal excluding allergy (0.4 %) and refracture (0.4 %)

The majority of the removal operations (74 %) was performed as an inpatient procedure and in 61 % during the first year after the initial operation (Table 1). Ankle (21 %) and wrist (15 %) were the most frequent anatomical sites of surgical implant removal (Fig. 1). Exact information on the type of implant and the different anatomical sites is shown in Table 2. Subjective, patient reported peri-/postoperative complications occurred in 33 patients (10 %). Fig. 2 illustrates that an impaired wound healing was the most common complication in 36 % followed by infections. A doctor’s recommendation was stated as the most common reason why patients opted for surgery (Fig. 3).

**Table 1:** Period from initial operation to implant removal

Time of removal after the initial operation	Portion
<6 month	33 %
7-12 month	28 %
13-18 month	20 %
19-24 month	11 %
>24 month	9 %

**Table 2:** Localizations and type of hardware

Type of material	Portion														
	Wrist	Ankle	Femur	Pelvis	Spine	Calcaneus	Middle and proximal Tibia	Clavicle	Talus	Upper arm	Foot	Middle Hand	Patella	Ulna without wrist	Radius without wrist
Wires	47 %	3 %	2 %			20 %		19 %	33 %	32 %	42 %	79 %			100 %
Plates	49 %		18 %	40 %		60 %	38 %	31 %		43 %	26 %	21 %		17 %	
Screws		36 %	36 %	60 %		20 %	21 %		66 %	18 %	32 %				
Screws and Wires	4 %												100 %	83 %	
Plate and Screws		53 %													
Intrame-dular nails		4 %													
Nail			43 %				41 %			7 %					
Internal fixator					100 %										
Prevot nails								50 %

96 (95 %) of 101 patients who had undergone the removal procedure due to pain and 72 (72 %) of the 100 patients with impaired function reported a subjective postoperative improvement concerning pain or function respectively.

Altogether, 52 % of the patients reported a subjective improvement of pain no matter what was the reason for operation. The same percentage (52 %) of all patients described an improved function after the operation.

**Fig. 4:**
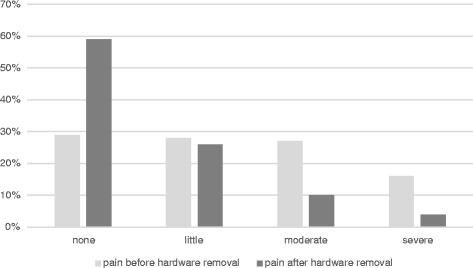
Occurrence of pain before and after surgical hardware removal

**Fig. 5:**
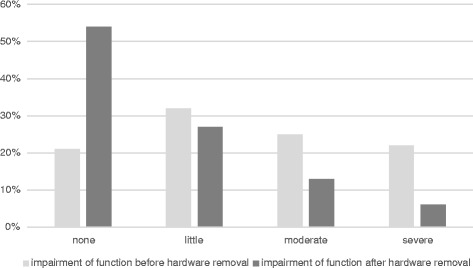
Rate of impairment of function before and after surgical hardware removal

Furthermore, 42 % of patients felt no change in pain before and after the operation, even though the procedure was successful and without complications. 7 % of patients without pain before the removal complained about pain afterwards. 5 % of the patients with pre-operative pain reported worsening of the pain following the removal procedure. An overview on pain and functional status before and after the removal is given in Figs. 4 and 5.

Finally, 96 % of patients stated, that they would opt for hardware removal again. 66 % of patients who suffered a complication would again decide to have the surgery done. If an (“absolute”) indication such as surgical site infection, metal allergy, soft tissue compromise, nonunion or failure of the osteosynthesis was the reason for hardware removal, 92 % of the responding patients would undergo surgery all over again. In case of a more relative indication such as the hope to improve function, foreign body sensations, a possible interference with a potential future procedure, pain or the patient’s desire for hardware removal, 97 % would have the procedure performed again. In the subpopulation of 101 patients who personally wished to have the implant removed as one reason for the operation, all of the patients would retrospectively decide to have the surgery done again independent of a subsequent subjective complication (Table 3).

**Table 3:** Percentages of patient groups who would undergo operation again related to indication of hardware removal

Over all	96 %
Absolute indications	92 %
Relative indications	97 %
Patients personal preference	100 %
Patients who suffered complications	66 %

Table 4 shows the specific results concerning ankle and wrist, being the most common location of implant removals.

**Table 4:** Implant removal on wrist and ankle

	Wrist	Ankle
Indication		
Pain	23 %	35 %
Impairment of function	29 %	36 %
Foreign body sensation	17 %	36 %
Allergy	0 %	1 %
Fear of cancer	0 %	0 %
Problems with metal detectors	2 %	0 %
Refracture	0 %	0 %
Malposition of the metal	13 %	7 %
Nonunion of the fracture (pseudarthrosis), insufficient stabilization of the fracture (failure of osteosynthesis)	0 %	14 %
Professional recommendation	67 %	64 %
Personal preference	33 %	46 %
Complication		
Re-fracture	0 %	0 %
Nerve damage	4 %	4 %
Infection	0 %	4 %
Impaired wound healing	0 %	11 %
Too much scare tissue (keloid development)	0 %	0 %
Bleeding	0 %	0 %
Thrombosis	0 %	0 %
Incomplete removal	0 %	4 %
Decision to opt for surgery again	98 %	93 %
	Wrist	Ankle
Pain		
Better	42 %	53 %
Same	52 %	39 %
Worse	6 %	8 %
Function		
Better	63 %	55 %
Same	35 %	45 %
Worse	2 %	0 %

## Discussion

To our knowledge this is the first survey assessing the patients’ individual experiences regarding surgical implant removal. Principle findings of this study were, firstly, that 10 % of the 332 responding patients who underwent orthopedic implant removal perceived complications occurred during or after the procedure with the most common complication being impaired wound healing. Secondly, when the indication for hardware removals was pain or limited function, patients reported a subjective improvement in 95 % and 72 % respectively. Thirdly, overall 96 % of all patients and even 66 % of the patients with peri- or postoperative complications would opt for the operation again. All of the patients who personally wished to have the implant removed would come to the same decision all over again even if they perceived having suffered complications. These results seem to contradict our initial hypothesis.

Several limitations must be considered regarding this study. The retrospective, open nature of the selection of the patients might result in bias, mainly, because not all of the patients who had surgical hardware removed in the observed time period were accessible for inclusion into this study. Concerning the response rate, similarly designed studies reached similar response rates [13, 14].

Particularly, results on reasons for the operation and the subjective satisfaction after the operation could be biased. This also holds true for “doctor’s recommendation”. This questionnaire item was not specified further; in our personal clinical experience as a specification of the German medical system, many patients present for implant removal because their general practitioner or orthopedic out-patient specialist without surgical capacity recommended to get the implants removed without further elaboration.

Furthermore, the contribution of a placebo effect cannot ultimately be excluded, because of the lack of a control group. Various orthopedic implant removals were assessed unrelated to the type of implant, anatomical site or mode of previous surgical implant application, which makes the analyzed population somewhat heterogeneous. Finally, our observations are based on pure subjective patient information, even for type and severity of complications, for pain and function in a non-validated questionnaire. Therefore our results may only carefully be compared to more objective studies based on physical examination and standardized outcome measures or specific scientific scores.

However, we deliberately chose this study design as the principle goal of this study was to assess the individual and subjective impression of the affected patients themselves. Due to their design and make orthopedic implants may permanently remain inside the body. Out of this reason and the often elective nature of the intervention, patients’ consent and request for the implant removal is central to the entire procedure. In order to analyze this, the personal impressions of the included patients themselves is what first and foremost can contribute to the assessment of the patients’ quality of life and level of satisfaction after undergoing surgery. And thereby, from our point of view, patient satisfaction and patients’ perception of the success of the treatment are among the most important goals for a successful surgical practice.

### Complications

Accurate data on peri-/postoperative complications due to surgical hardware removal is currently scarce. This accounts for complication rates of orthopedic fracture fixation itself, too. Furthermore the documentation of complications varies with different study design. Importantly, our data are based on complications perceived by the patients themselves and a comparison with other studies has to be done with precaution. Depending on the reporting source, complication rates of radial palmar plate osteosynthesis seem to differ between less than 5 % and up to 27 % [15–19]. In surgically treated ankle fractures, complication rates were documented in 5 % of the cases [20]. Operatively managed clavicle fractures revealed complications in 5 to 15 % [21]. Complication rates for other frequently performed operations are 1.6 % following standard knee arthroscopies [22] and 10 % following intervertebral disc surgeries [23].

In literature complication rates of surgical hardware removal are heterogeneous and reach from 0 to 40 %. Complication rates and kinds (refractures, wound infections or nerve damages) differ with various implants and body sides. Furthermore documentation of the complications and study design are heterogeneous [7, 9–12, 24, 25].

The overall complication rate of 10 % in the assessed group of patients in our study with above mentioned limitations corresponds well to the existing data. In contrast, wound healing problems seem to occur more frequently in our group (36 % of complications) than in the previously described studies. However, the fact that our patients judged themselves on the impairment of wound healing (as opposed to assessment by healthcare professionals in other studies) renders this discrepancy somewhat less significant. Furthermore, we report surprisingly low rates of postoperative re-fracture (7 % of complications) and nerve damage (14 % of complications) which both were described to occur more frequently in previous studies [5]. Comparing the complication rates of hardware removal from our observations to the initial hardware implantation surgery as well as to other commonly performed surgeries, the complication rate does not seem remarkably different.

### Improvement of pain and function after implant removal

Our data reveal a high percentage of subjective improvement of pain after implant removal. Regarding all of the analyzed patients, 52 % stated an improvement. If undergoing the operation due to pain 96 % described less pain after the operation. In literature, improvement rates concerning pain are heterogeneous. One study of ankle plate removal showed an improvement of 50 % [26]. Other reported data in intramedullary nail removal of the tibia and the femur could detect advancements in 64 % to 96 % [9, 27]. Specifications for the exact reason of implant removal are not given in all of the studies.

Concerning function, in our study similar improvement rates (52 %) were seen as in pain if evaluating the entire patient population without regarding the different reasons why the surgery was performed. In the subpopulation where impairment of function was a reason for operation, the improvement rates were higher (72 %). This is in good accordance with a study by Miller who reported a subjective and objective improvement of function after syndesmotic screws and ankle plates were removed for all patients included in the study (25 patients) [28]. In contrast a sole removal of syndesmotic screws showed postoperative no better ankle function as in a group with retained screw fixation [29]. Alike our data with a comparable choice of body sides but only 60 patients Minkowitz’ observed an improvement of function of 44 % 1 year after the implant removal was observed [25].

Comparing existing works to our data we included a larger patient population with a wider variability of body regions of hardware removal. However if as well plates are removed, existing data support our results: removal of implants is a good option to improve pain and impairment of function after orthopedic surgery.

### Individual satisfaction after surgical implant removal

Surprisingly, 96 % of all patients, and even 66 % of the patients who subjectively perceived complications after hardware removal would opt for surgical implant removal again. To date, there is very sparse, if any published information on patients’ satisfaction after undergoing surgical hardware removal. Our results indicate, that patients are content to a surprisingly high degree after implant removal, particularly if their own personal desire was a reason for the operation. Taking into account the data we present in this study, it seems that the potential disadvantages such as postoperative complications are overcome by the factor of having foreign material removed from one’s own body. We associate the high satisfaction rate to this assumption, which is in accordance with every day work impression when interviewing patients regarding their view on implant removal. One may speculate in the light of the presented data, that at least the subjective need to have the implant removed ought to be a minimal requirement for the indication for implant removal. Vice versa, individuals without any complaints about the implant *in situ* may not be suited for this operation. This is in line with the findings of Gosling et al., who described that 20 % of asymptomatic individuals after femoral intramedullary nailing showed increased pain after surgical implant removal [30]. They concluded that only patients suffering from pain after femoral nailing would benefit from implant removal [30].

## Conclusion

In this patient survey, we report a surprisingly high rate of satisfied patients after surgical hardware removal. This may lead to the conclusion that implants should be removed by default. However, postoperative complications occurred at a rate of 10 %. Hence, for the sake of both patients’ safety and quality of life, the indication for hardware removal still has to be assessed with scrutiny. Nevertheless, removal of implants might relief pain, increase range of motion and function and thus enhance the patient’s satisfaction. The definite causality between psychological factors, satisfaction and physiological improvement needs further investigations.

## Additional file

Additional file 1:
**Questionnaire translated in English.** (DOCX 23 kb)
