# Supplementary material for: Metal implant removal: benefits and drawbacks – a patient survey
Source: BMC Surg. 2015 Aug 7;15:96. doi: 10.1186/s12893-015-0081-6 (PMC4528685; doi:10.1186/s12893-015-0081-6)
Supplement: Additional file 1: — Questionnaire translated in English. (DOCX 23 kb) [file 12893_2015_81_MOESM1_ESM.docx]

**1. What was the reason for the implant removal?**

(Multiple selections are possible)

- pain
- impairment of function
- foreign body sensation
- allergy
- fear of cancer
- problems with metal detectors
- refracture
- malposition of the metal
- nonunion of the fracture (pseudarthrosis), insufficient stabilization of the fracture (failure of osteosynthesis)
- professional recommendation
- personal preference

**2. Did complications occur after the surgery?**

○ No ○ Yes ○ not applicable

If yes, what was the complication?

(Multiple selections are possible)

- Re-fracture
- Nerve damage
- Infection
- Impaired wound healing
- Too much scare tissue (keloid development)
- bleeding
- thrombosis
- incomplete removal
- other: _________________

**3. What time after the fracture the metal was removed?**

○ up to 6 months ○ 7-12 months ○ 13-18 months ○ 19-24 months ○ over 24 months

**4. How long lasted the absence from work after the operation:**  ____ days

**5. Did you suffer from pain before the metal removal?**

○ no pain ○ little pain ○ moderate pain ○ severe pain

**6. Did you suffer from pain after the metal removal?**

○ no pain ○ little pain ○ moderate pain ○ severe pain

**7. Was the function impaired before the metal removal?**

- No impairment ○ little impairment
- Moderate impairment ○ severe impairment

**8. Was the function impaired after the metal removal?**

- No impairment ○ little impairment
- Moderate impairment ○ severe impairment

**9. Today, would you decide again for an operation to remove the metal?**

○ No ○ Yes
